# New approaches for unravelling reassortment pathways

**DOI:** 10.1186/1471-2148-13-1

**Published:** 2013-01-01

**Authors:** Victoria Svinti, James A Cotton, James O McInerney

**Affiliations:** 1 Department of Biology, National University of Ireland at Maynooth, Maynooth, Co Kildare, Ireland; 2 Current address: Wellcome Trust Sanger Institute, Wellcome Trust Genome Campus, Hinxton, Cambridge, CB10 1SA, UK; 3 Current address: Department of Microbiology & Immunology, Life Sciences Centre, University of British Columbia, Vancouver, BC, V6T 1Z3, Canada

## Abstract

**Background:**

Every year the human population encounters epidemic outbreaks of influenza, and history reveals recurring pandemics that have had devastating consequences. The current work focuses on the development of a robust algorithm for detecting influenza strains that have a composite genomic architecture. These influenza subtypes can be generated through a reassortment process, whereby a virus can inherit gene segments from two different types of influenza particles during replication. Reassortant strains are often not immediately recognised by the adaptive immune system of the hosts and hence may be the source of pandemic outbreaks. Owing to their importance in public health and their infectious ability, it is essential to identify reassortant influenza strains in order to understand the evolution of this virus and describe reassortment pathways that may be biased towards particular viral segments. Phylogenetic methods have been used traditionally to identify reassortant viruses. In many studies up to now, the assumption has been that if two phylogenetic trees differ, it is because reassortment has caused them to be different. While phylogenetic incongruence may be caused by real differences in evolutionary history, it can also be the result of phylogenetic error. Therefore, we wish to develop a method for distinguishing between topological inconsistency that is due to confounding effects and topological inconsistency that is due to reassortment.

**Results:**

The current work describes the implementation of two approaches for robustly identifying reassortment events. The algorithms rest on the idea of significance of difference between phylogenetic trees or phylogenetic tree sets, and subtree pruning and regrafting operations, which mimic the effect of reassortment on tree topologies. The first method is based on a maximum likelihood (ML) framework (*MLreassort*) and the second implements a Bayesian approach (*Breassort*) for reassortment detection. We focus on reassortment events that are found by both methods. We test both methods on a simulated dataset and on a small collection of real viral data isolated in Hong Kong in 1999.

**Conclusions:**

The nature of segmented viral genomes present many challenges with respect to disease. The algorithms developed here can effectively identify reassortment events in small viral datasets and can be applied not only to influenza but also to other segmented viruses. Owing to computational demands of comparing tree topologies, further development in this area is necessary to allow their application to larger datasets.

## Background

Influenza viruses are a major cause of infections in humans, with a dynamic history characterized by common seasonal epidemics and occasional pandemics. The evolution of the virus during and in between these outbreaks is difficult to describe because it undergoes rapid evolution in order to evade the constantly adapting immune response of their hosts [1]. The influenza A genome consists of eight individual segments of single stranded, negative sense RNA, each containing a single gene [2,3]. The segmented nature of the genome allows for the exchange of entire genes between different viral strains when they co-infect the same cell through the process of reassortment [4]. Two viruses co-infecting a cell could potentially generate 254 genotypes. Reassortment is particularly interesting because it is an event that can quickly generate influenza strains with novel infectious properties [5-7]. The most recent pandemic in 2009 provides a fresh example of a reassortant H1N1 strain that arose to claim an infectious ability of global scale [8]. In fact, three of the most recent pandemics have been caused by reassortant influenza A strains (1957, 1968 and 2009) [9]. Li and colleagues generated all the possible reassortants from two H5N1 and H3N2 strains and found that 72% of all the new subtypes replicate differently to the parental strains whereas 28% were not viable [10]. In addition, 22 reassortants were more pathogenic in mice than the parental H5N1. These results underline the importance that reassortment plays in the evolution of influenza viruses and how essential it is to be able to describe these events. Identification of new reassortants is a crucial step in understanding viral evolution and preferential reassortment patterns, in working towards preventing infections with and spread of fatal viruses.

Phylogenetic trees depict hypotheses of evolutionary relationships between species or sequences [11]. If no reassortment events have occurred in the history of a set of sequences, then, allowing for phylogenetic error and stochastic effects, we expect that their respective phylogenetic trees will display overall congruence. More formally stated, we have a null hypothesis that a single phylogenetic tree (i.e. evolutionary history) best fits all regions of the genome. The alternative hypothesis is that some part of the genome fits an alternative phylogenetic tree better and this is sufficient to overcome our initial expectation of a single tree - thereby rejecting the null hypothesis in frequentist terms, or overcoming our prior in Bayesian terms. Reassortment events, therefore, can be identified in those cases where genetic segments from the same isolate occupy different positions on the phylogenetic trees inferred from these segments [12]. In many studies up to now, the assumption has been made that if two phylogenetic trees differ, it is because reassortment has caused them to be different [13-16]. While phylogenetic incongruence may be caused by real differences in evolutionary history, it can also be the result of evolutionary model misspecification [17], high levels of homoplasy [18], long branch attraction [19], inadequate sampling [20] or separating data into partitions [21]. Therefore, if we wish to understand the frequency and nature of reassortment we must have methods for distinguishing between topological inconsistency on phylogenetic trees that can be accounted for by confounding effects and topological inconsistency that is due to reassortment. A reassortment event is more easily detected if the two strains involved in producing the reassortant are sufficiently divergent in their sequences. Reassortment between very similar strains is likely to go undetected by most, if not all, methods. At its most extreme, consider the instance where reassortment has involved two identical parental strains. This event cannot be detected using a computational approach but it is also not relevant in a biological context, as we would expect the identical gene segments to have identical properties. In addition to the detection of the existence of reassortment events, it is desirable to be able to identify the direction of reassortment – the likely origin of genetic segments in a reassortant strain - and also the frequency with which particular segments reassort. This information can be translated into an understanding of overall reassortment pathways and can be used to make predictions about future reassortment events.

In order to accomplish the objective of adequately reconstructing reassortment events in influenza, it is necessary to have a robust statistical platform, based on evaluating the differences between phylogenetic trees derived from different viral genome segments. In this paper we assess reassortment by the evaluation of the significance of the differences between phylogenetic trees constructed from different genome segments. If phylogenetic trees constructed from distinct segments are non-trivially different (in other words, if the differences in tree topologies cannot be explained by stochastic effects, by having insufficient amount of data, low levels of phylogenetic signal or high levels of artifactual noise), the occurrence of one or more reassortment events may be the best explanation of this divergence. If the differences between phylogenetic trees are trivial, say, when they can be accounted for by invoking error in tree estimation as an explanation, it is appropriate to assume that no reassortment has taken place. We acknowledge however, that even though we might reject the null hypothesis (no reassortment), there can be situations where this is not correct. We do not have a method for discovering the false rejection rate and indeed it is unlikely to be discoverable using current technology.

The Robinson-Foulds (RF) distance [22] and subtree transfer distances (subtree pruning and regrafting, or SPR) [23] are two methods of measuring dissimilarity when comparing trees. Counting how many bipartitions in one tree are not shared with the other tree gives a value of the RF distance between two trees. However, this metric treats each feature of the tree equally and as a result, trees that agree in an important area can be given a large tree distance [11]. An SPR operation on a tree consists of pruning a subtree by cutting an edge, and then regrafting the subtree by the same cut edge to a new vertex [23]. It can be said that two trees are close together if one can be obtained from the other by a small number of SPR operations [24]. The “cut and paste” nature of this operation resembles the effect caused by reassortment events. Therefore, identifying the minimum number of SPR operations to convert one phylogenetic tree into another can describe the minimum number of reassortment events that have occurred in the history of the strains being studied. The EEEP (Efficient Evaluation of Edit Paths) [25] algorithm can be used to achieve this objective in a very short time. EEEP works in a pairwise manner: it uses a reference and a test tree to construct the ‘edit path’ between them, or the SPR operations needed to convert the reference tree to the test tree. Choosing this fast algorithm for the calculation of edit paths resulted in a pairwise approach to the reassortment problem, the outcomes of which are combined at a later stage in the analysis.

Not all differences in phylogenetic tree topology need to invoke reassortment events as the explanation. Determining whether the topologies produced by SPR modifications are significantly different to the starting tree, requires a statistical model for the variation of the features of the tree [11]. Tests such as the bootstrap probability [26], Kishino-Hasegawa [27], and the approximately unbiased (AU) test [28] are used to assess confidence in phylogenetic hypotheses. In order to determine whether trees represent significantly different hypotheses based on a particular dataset, the AU test has been used [29-31]. This test uses a multi-scale bootstrap technique that consists of generating sets of bootstrap replicates with varying sequence lengths and estimating the AU p-value from the change in the bootstrap probability values along the changing sequence length [28]. Given a set of trees and an alignment of sequences, the resulting p-value for each tree reflects how well that particular topology describes the given data. The AU test has some desirable properties. The use of an explicit model of sequence evolution in order to reconstruct the history of a segment of DNA is an important step in understanding virus evolution. In addition, the AU test has a robust statistical platform on which to evaluate the significance of the difference between sets of phylogenetic trees. In our analyses, we have used the AU test for the sets of trees produced by the EEEP program for each pairwise analysis.

We present two automated methods to assess the significance of difference between phylogenetic trees and we use these methods to test whether reassortment occurred in a simulated dataset as well as a small collection of influenza viruses isolated in Hong Kong in 1999. The first method (*MLreassort*) applies subtree pruning and regrafting (SPR) modifications on maximum likelihood trees and the AU test to identify significant differences in tree topologies. The second method (*Breassort*) uses a Bayesian approach, together with multidimensional scaling and SPR, to identify the most frequent SPR operations that connect two significantly different sets of trees. Reconciliation of topologies is sought between all pairs of maximum likelihood trees derived from the eight influenza genetic segments. The steps required to reconcile two trees, starting with one tree (the reference tree) and perturbing its topology to obtain the other tree (the test tree), involve subtree pruning and regrafting operations. We only infer reassortment events for those SPRs that are found by both methods as a conservative approach. Although one method may be found to perform better than the other, for now we have opted to focus on consistency across methods. We propose this kind of approach as a way of evaluating the nature of reassortment in influenza and other segmented viruses.

## Results and discussion

Reassortment is a process that results in conflicting phylogenetic hypotheses derived from their respective gene segments. Our overall aim is to detect real reassortment events that have occurred in a set of influenza isolates. We apply these algorithms to a small set of influenza sequences as well as a simulated data set. The enormous complexity of influenza virus evolutionary history cannot reasonably be explored in this manuscript, so instead we present exemplar datasets and describe how the approaches work. We also suggest future improvements that would be useful for a thorough understanding of the complex nature of reassortment networks.

### Simulated data

**Figure 1:**
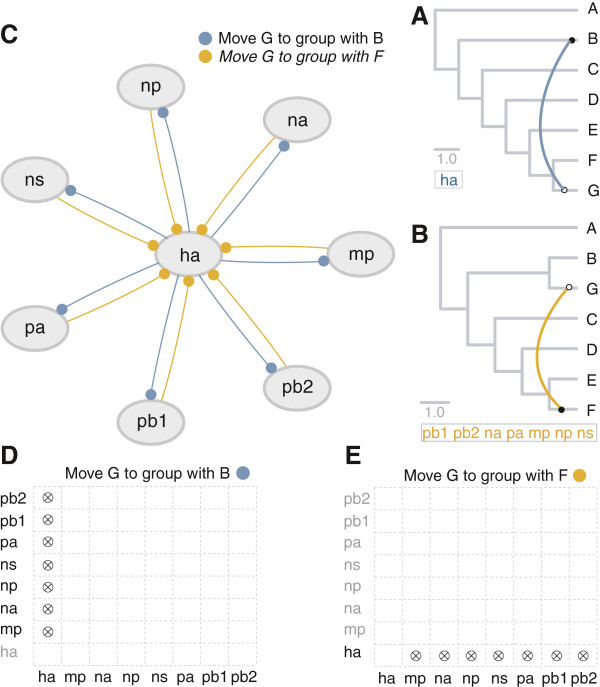
**Simulated data with manually introduced SPR modifications.** The HA data was simulated on a tree modified by moving taxon ‘G’ to group with taxon ‘B’. (**A**) Output from the ML analysis for seven segments: MP, NA, NP, NS, PA, PB1 and PB2. A significant SPR was detected that would require moving taxon ‘G’ to group with taxon ‘B’, as suggested by the HA segment (direction of arc from empty to filled circle). Colours of arcs correspond to specific SPR operations. (**B**) HA tree: seven segments propose a significant SPR modification on the HA tree that would require moving taxon ‘G’ to group with taxon ‘F’. (**C**) Frequency network from Bayesian results. Edges point from segment proposing an SPR, to the segment whose tree needs to be modified (filled circle). Legend shows SPRs corresponding to the coloured edges. HA proposes moving taxon ‘G’ to group with taxon ‘B’ for the other seven segments. Conversely, the rest of the segments suggest that ‘G’ should move to cluster with ‘F’ on the HA tree. (**D**), (**E**) Overlap between *MLreassort* and *Breassort*. The *x*-axis represents the segments that propose the SPR move, whereas the *y*-axis represents the segments whose trees need to be modified according to that SPR. The name of a tree segment is greyed out in the case where the SPR move is irrelevant, i.e. when the taxa involved in the move are sister-taxa.

In order to test the ability of our methods to identify manually induced reassortment events, we carried out a straightforward analysis using simulated data (Figure 1). Eight alignments, each containing seven sequences, were simulated under a common phylogenetic hypothesis (see methods for details). The lengths of these alignments corresponded to the lengths of the eight influenza A virus segments. In order to induce a situation where reassortment is imitated, the HA alignment was then simulated again, this time using a different phylogenetic tree topology. This hypothesis specifically contained a manually introduced reassortment event involving taxon ‘G’.

We inferred phylogenetic trees for all the eight segments using the GTR model of evolution. For *MLreassort*, maximum likelihood trees were obtained for each segment (Figure 1A and B). In order to evaluate the significance of the differences between pairs of maximum likelihood tree topologies, we used the EEEP (Efficient Evaluation of Edit Paths) algorithm [25] in combination with the AU test to first of all define the best way of using SPR edits to convert one tree into another via the fewest moves and subsequently to evaluate whether any of these edits result in statistically significant changes in tree topology. Once the SPR edits and resulting tree topologies are obtained, the AU test is used to estimate confidence intervals around trees. Non-overlapping confidence intervals indicate that the trees included in the sets are significantly different to each other. SPR operations connecting trees with significantly different topologies are referred to as significant operations/transitions/branch swaps, and they are depicted on the maximum likelihood tree for each segment (Figure 1A and B). The direction of arcs, pointing from the end with an empty circle (the likely source) to filled circle (the recipient lineage) indicates the direction of transfer. For the simulated data, all significant branch swaps involved taxon ‘G’. Using the HA alignment (the alignment simulated using a reassortment-containing hypothesis) and the GTR model of evolution, a HA tree was obtained (Figure 1B) with a negative log likelihood of 9256.28. *MLreassort* proposes a branch swap on the HA tree to reconcile it with the other seven trees (moving taxon ‘G’ to group with taxon ‘F’). After applying this SPR to the HA tree, the negative log likelihood increases by 1868.43 (to 11124.71). This means that the tree obtained after the SPR modification provides a worse explanation of the HA data than the tree before the SPR modification.

*Breassort* uses a Bayesian-based approach for tree reconstruction. The result of a Bayesian MCMC sampling of parameter space, including tree space, is a set of phylogenetic trees. In order to detect reassortment, we developed a method for comparing these sets. One way of achieving a meaningful comparison of two sets of trees that are derived from two different alignments is to evaluate tree ‘distances’, both within and between the two sets, in geometric space [32], with each point in this space representing a phylogenetic tree. Using a standard statistical method, convex hull peeling [33], we arranged the points into convex hulls. Eliminating the 5% outliers in each set of trees using convex hull peeling, we carried out pairwise comparisons of the remaining 95% of trees. The pairs of Bayesian tree sets all overlap, with the exception of cases in which HA is involved. The set of HA trees does not overlap with the sets of trees for MP, NA, NP, NS, PA, PB1 and PB2. This kind of situation might indicate that the genes have different evolutionary histories, but could also be caused by stochastic effects as might be expected, for instance, if the alignments were short. Therefore, for those cases where the sets of trees do not overlap, we randomly selected 100 trees from each segment’s tree space. We then carried out EEEP analyses in order to find branch swaps that connect the trees in the two sets. SPR edits that were found in more than 70% of cases were considered significant. The results of these comparisons are summarized using a network diagram, where each node represents the set of trees from an MCMC Bayesian analysis for its corresponding segment (Figure 1C). The edges between the nodes correspond to the proposed SPR modifications, and the direction (ends with filled circles) is towards the trees being modified. Edges of the same colour show the same SPR operation, as shown in the legend.

In inferring reassortment events, we have opted for a conservative approach that uses consistency across both *MLreassort* and *Breassort* (Figure 1D and E). The HA tree proposes that all the other seven trees should be altered by moving taxon ‘G’ to cluster with taxon ‘B’ (Figure 1A, C and D). On the other hand, segments MP, NA, NP, NS, PA, PB1 and PB2 propose that an SPR transition on the HA tree, which would involve moving taxon ‘G’ to group with taxon ‘F’ (Figure 1B, C and E), is required in order to reconcile the trees. This modification would result in a topology that would resemble the original HA tree, before the manual introduction of the modification. Interpreting the output in the light of all the results, it makes sense to infer that the HA tree, rather than seven other trees, has a ‘wrong’ topology. This would imply that taxon ‘G’ has an HA segment that is inconsistent in its evolution with the other seven genes. If this was a real viral data it would mean that ‘G’ is a reassortant strain that has acquired a HA segment from a ‘B’-like strain. Therefore, the SPR move manually introduced into the HA tree by moving taxon ‘G’ to group with ‘B’ (instead of ‘F’), has been recovered by both algorithms. This result suggests that the algorithm described can accurately detect manually induced reassortment events. This is not a stringent test of the methods and more complex scenarios such as those involving multiple reassortant viruses will be more difficult to detect, however, the approach can also be shown to work well with small datasets of real viral data.

### Real data

Nucleotide alignments were obtained for influenza A sequences isolated in Hong Kong in 1999 and whose lengths ranged from 890 aligned positions for NS to 2,341 positions for PB1 and PB2 (Table 1). First we carried out a number of analyses in order to assess whether the data were suitable for phylogenetic analysis using standard phylogenetic methods. All alignments passed the permutation tail probability (PTP) test [34] (Table 1). Likelihood mapping analyses [35] showed that over 94% of quartets are fully resolved (data not shown). The test for intra-segment recombination (see methods) produced p-values that range from 0.016 to 0.96. At p-value cut off of 0.05, the null hypothesis (that there is no recombination) is not rejected by most of the segments, except for NS (Table 1). However, before eliminating three of the four near identical (≥99%) environmental sequences isolated in 1999, the p-value for the alignment was 0.22, well above the threshold level. This segment passes other recombination tests such as NSS and MaxChi^2^ and therefore we think that the lower Phi p-value for the NS is likely to be due to stochastic effects. Given that the data seems to be amenable to phylogenetic analysis, we conducted an analysis of reassortment. In order to construct phylogenetic trees for each gene, a model that best describes the evolution of the sequences was selected. We evaluated compositionally heterogeneous models, implemented in p4 [36] and found that they did not significantly improve the likelihood of the data (not shown) and therefore, we carried out all analyses using compositionally homogeneous models. The homogeneous models of nucleotide substitution (GTR and TVM) and the maximum likelihood scores for the best trees (ranging from 10147.76 to 2496.26) are shown in Table 1. The strain A/redknot/NJ/325/1989 H7N7 was used as the outgroup in all phylogenetic trees.

**Table 1:** Alignments and models for Hong Kong 1999 dataset

**Segment**	**Alignment length (nt)**	**PTP test p-value**	**PHI test p-value**	**Model selected**	**Best tree (−ln L)**
HA	1862	0.01	9.11e-02	GTR+I+G	10147.76
MP	1034	0.01	4.78e-01	TVM+G	2496.26
NA	1529	0.01	6.86e-01	GTR+I	6900.64
NP	1565	0.01	8.24e-01	GTR+G	3744.44
NS	890	0.01	1.61e-02	TVM+I	2761.72
PA	2233	0.01	3.39e-01	GTR+I	5678.98
PB1	2341	0.01	9.88e-01	GTR+G	5856.48
PB2	2341	0.01	1.98e-01	GTR+G	6573.47

**Figure 2:**
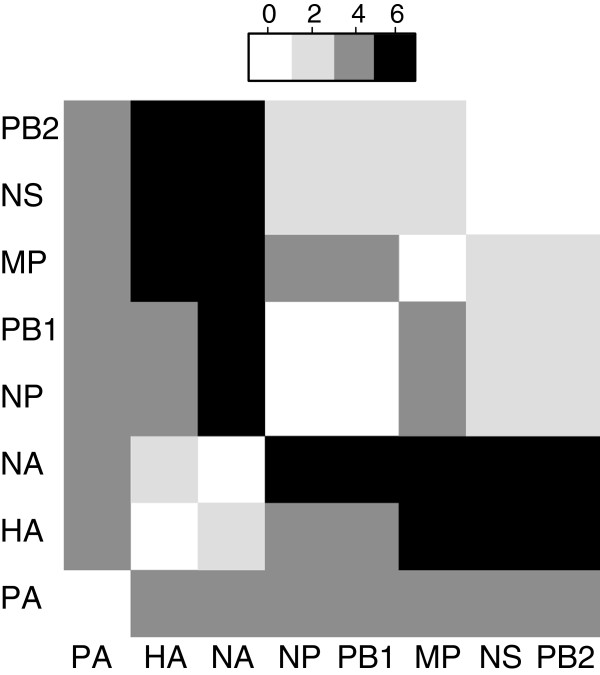
**Robinson-Foulds distances between trees of the Hong Kong dataset.** The intensity of the squares corresponds to the degree of distance. Distances range from 0 to 6, representing the number of bipartitions present in one tree but not in the other. Some trees have the same topology (NP and PB1, NS and PB2) whereas the NA tree seems to be most distant to the other trees (distance of 6).

The Robinson-Foulds (RF) distance used to carry out an initial analysis of the extent to which the maximum likelihood tree topologies for each segment were different (Figure 2). This test says nothing about the significance of the differences between these trees; it is simply a measure of the degree to which they have bipartitions in common. The number of partitions present in one tree but not in another range from 0 (PB1 and NP have the same topology, and PB2 has the same topology as NS) to 6 (8/28 pairs have an RF distance of 6). For those trees that manifested conflict, we evaluated the shortest edit paths required in order to reconcile their topologies, using the EEEP algorithm and the AU test (see methods). As outlined earlier, these rearrangements mimic reassortment events. In total, 56 pairwise comparisons of tree topologies were carried out. Four of these cases involved identical trees making any inference of reassortment unnecessary. The confidence intervals around trees, estimated using the AU test, overlap in twenty-four cases. While we have done many independent (pairwise) tests, the number of trees in each test is quite small (ranging from 3 to16 trees for each calculation of AU p values). We have corrected these p values using the Benjamini and Hochberg [37] test (BH test) and note that the correction has little impact on the resulting confidence intervals (data not shown). This means that for these 24 tree pairs, the topological differences can be accounted for by stochastic errors; 46% (26/56) of cases with non-overlapping confidence regions remained. Significant edits (SPR operations that result in a significant change in likelihood score) between trees with non-overlapping confidence regions were determined and depicted on each ML tree (shown in Additional file 1: Figure S1). In the cases where the arcs are bidirectional, the source and recipient taxa cannot be determined with certainty. Bayesian phylogenetics (*Breassort*) was also used to infer the evolutionary histories of each segment, by carrying out 28 pairwise comparisons of sets of trees using 95% confidence intervals (as described in methods). In 57% (16 out of 28) of cases the two sets of trees do not overlap, which indicates that the trees in one set cannot be used to explain the data from which the trees in the other set are derived. The analysis was repeated using different thresholds (90%, 95%, 99%, 99.9%, 3 replicates each) for determining confidence intervals (Additional file 1: Figure S3). Small variations in the networks are expected to occur due to the reduction of multi-dimensional space to 2D, and the arbitrary choice of trees to compare from each convex hull. However, consistent signals were identified irrespective of the analyses, and variations between different CI thresholds are not greater than those observed when repeating the analysis with the same parameters. The findings from both algorithms are discussed below, starting with the example of a specific pair of segments: NS (non-structural) and NA (neuraminidase).

**Figure 3:**
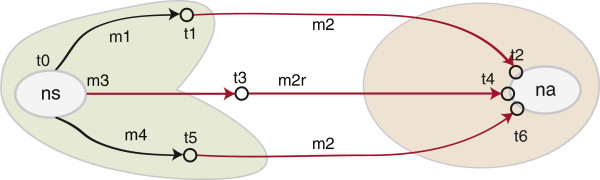
**Trees NS and NA, confidence intervals and SPR modifications.** Subtree pruning and regrafting (SPR) modifications that the NA tree proposes on the NS tree, and the confidence interval around each tree (coloured shapes). Three paths are possible. The labels on the arrows refer to nodes involved in a move: m1 - move outgroup to cluster with hk1774, m2 – move hk1073 to cluster with hk1774, m2r – reverse of m2, m3 – move hk1073 to cluster with outgroup, m4 – move env99 to cluster with quail99/sh39/hk1073 group. t1 - t6 are trees resulting from applying these SPR modifications to the NS tree. Arrows between two trees in the same confidence interval (CI) reflect trivial differences (e.g. m1, black arrow), whereas ones between trees from different CIs are considered significant (e.g. m2, red arrow). We consider m2 as significant as we’re interested in the minimum amount of significant branch moves between NS and NA.

**Figure 4:**
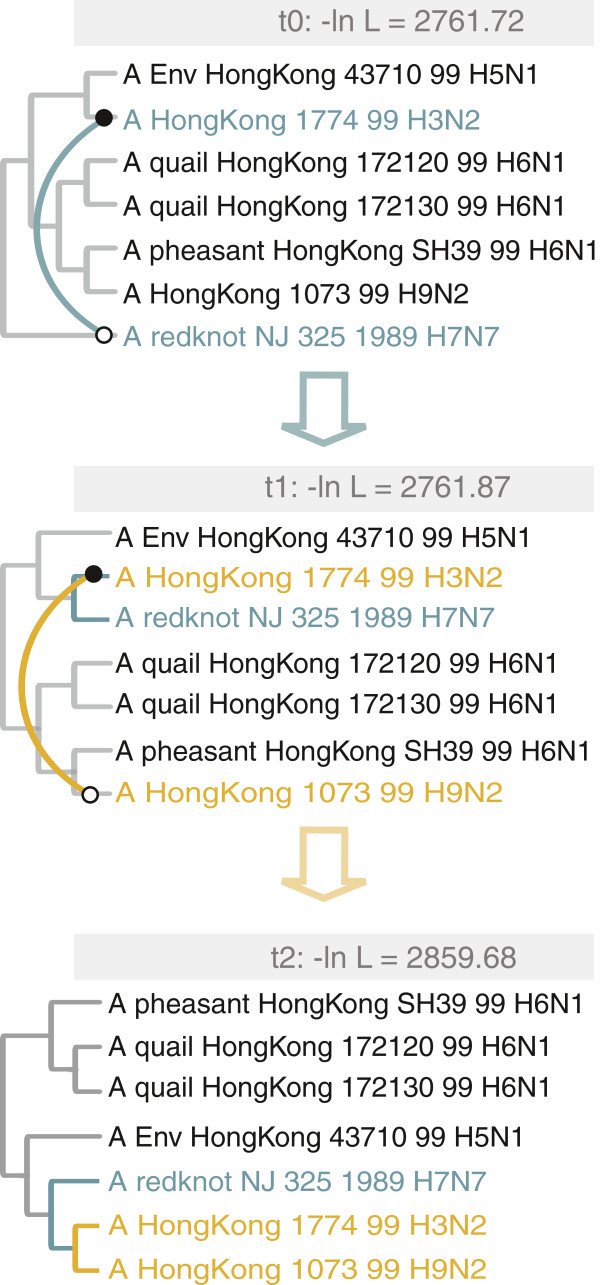
**Shortest edit path between NS and NA.** Trees depicting the *m1*, *m2* SPR edits from Figure 3. Arc direction is from empty to filled circle. NS (*t0*) is the start tree (−ln L = 2761.72), on which we apply modification *m1* (moving A/redknot/NJ/325/1989 H7N7 to A/HongKong/1774/99 H3N2). The log likelihood of the intermediate tree (*t1*, -ln L = 2761.87) is close to that of NS (this tree is inside NS’ CI). The second modification, *m2*, is applied to *t1* and results in a topology identical to that of the NA tree (*t2,* -ln L = 2859.68). The rise in the negative log likelihood of the tree after *m2* is 97.81 (the resulting tree is outside NS’ CI).

**Figure 5:**
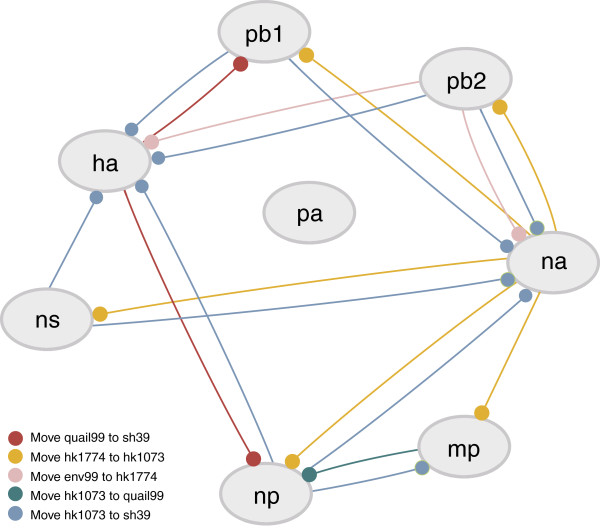
**Network of the most frequent SPRs from *Breassort. ***Each node represents the set of trees for the corresponding segment. Edge colours correspond to different types of SPR operations, as shown. Edges point from a segment that proposes the branch swap, to the one that needs to be modified (ending in filled circle). For example, the orange edge going from NA to NS depicts the following operation: cutting the branch leading to A/HongKong/1073/99 H9N2 and reconnecting it to the branch ending in A/HongKong/1774/99 H3N2. The NS tree is the one being modified, and the NA tree proposes this modification.

The models of evolution selected for the two datasets are TVM+I for NS and GTR+I [38,39] for NA (Table 1). For *MLreassort*, the RF distance between the resulting ML trees is six (Figure 2). Three equally optimal edit paths were found between the ML trees derived from the two segments (Figure 3). A minimum of two SPR operations is necessary to convert the NS tree into the NA tree and vice versa. Using the AU test, an hypothesis can be rejected if the difference in tree topology is not greater than might be expected by chance. The AU test therefore allowed us to estimate the confidence intervals for the NS and NA trees. The trees found inside NS’ confidence interval are not significantly different to NS (i.e. differences can be explained adequately by lack of power caused by finite data size), whereas the trees found outside this interval are significantly worse at explaining the NS data. That is, the difference between a tree found within the NS confidence set and one found outside this space is best explained by real differences in evolutionary history. This difference is described by an SPR operation that is considered significant (produces a significant difference in tree topology). Among the three calculated edit paths, a few significant SPR operations were found (*m2* and *m3*, *m2r*, Figure 3). We consider the shortest (*m2*) corresponding to moving taxon A/HongKong/1073/99 H9N2 (hk1073) and grouping it with taxon A/HongKong/1774/99 H3N2 (hk1774). The trees involved in this edit path are depicted in Figure 4. The start (NS) tree is shown together with the trees *t1* and *t2* resulting from applying the suggested SPR operations (*m1, m2*). It is important to note the increase in negative log likelihood after applying the second operation (*m2*) on the *t1* tree. The resulting tree (*t2*) is significantly different to the NS tree, as it is also illustrated in the confidence intervals diagram (Figure 3). The reassortment network resulting from *Breassort* depicts the move of taxon hk1073 to group with hk1774 by orange edges (Figure 5). It can be noted that some SPR proposals appear much more abundantly than others (e.g. orange versus pink edges).

**Figure 6:**
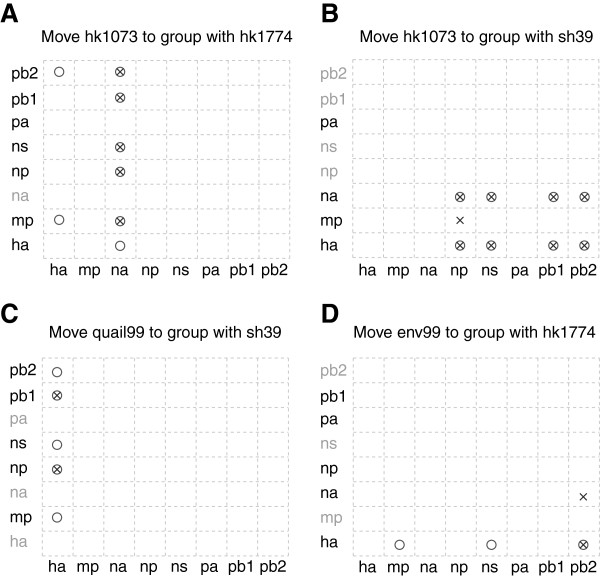
**Combined SPRs from *MLreassort *and *Breassort. ***Each plot depicts an SPR move, with symbols indicating the cases when this move is significant. Circles represent results from the maximum likelihood-based approach, while crosses represent results from applying the algorithm based on a Bayesian framework. The x-axis shows the segments that propose the specified SPR, while the y-axis shows the segments whose trees need to be modified. The name of a tree segment is greyed out in the case where the SPR move is irrelevant, i.e. when the taxa involved in the move are sister-taxa. For example, moving hk1073 to group with hk1774 in the NA tree is irrelevant, as the NA tree already has these grouping together.

Although it may be that one algorithm performs better than the other, for now we will concentrate on those SPR edits recovered by both algorithms (Figure 6). An SPR modification that involves clustering A/HongKong/1073/99 H9N2 (hk1073) with A/HongKong/1774/99 H3N2 (hk1774) was found by both algorithms (Figure 6A). This move causes a significant change in the likelihood value for the NS’ tree given the data (as seen in Figure 4) and is the topology that is proposed by the NA tree, where these strains group together. The two viruses possess an NA surface protein of type N2 (hk1774 is a H3N2 and hk1073 is a H9N2 strain), explaining why they cluster together in the NA tree. Since the NA tree pairs hk1073 and hk1774 together, it proposes this grouping for most of the other trees (except for PA) (Additional file 1: Figure S1). For the HA tree, either the hk1774 or hk1073 taxa can be moved (bi-directional), whereas for MP, NP, NS, PB1 and PB2, the hk1073 branch needs to be pruned and regrafted onto hk1774 in order to reconcile the topologies. In the network diagram, the NA segment proposes grouping hk1073 with hk1774 in MP, NP, NS, PB1 and PB2 (Figure 5, orange edge). Given that hk1073 is at the expected position in the NA tree, we must hypothesize that the evolutionary history of the other segments belonging to the H9N2 isolate is in conflict with that of NA. Therefore, hk1073 could be a reassortant strain that acquired the NA segment of an H3N2-like virus, and the rest of the segments from other sources.

Another significant SPR modification (Figure 6B) involves grouping hk1073 with A/pheasant/HongKong/SH39/99 H6N1 (sh39). A general trend can be seen where the majority of trees suggest this modification for the HA and NA trees. The topologies of the six internal segments show hk1073 (of type H9N2) clustering with the H6N1 group, whereas this is not the case with the two external viral proteins, HA (where the hk1073 sequence is placed as an outgroup to the H6N1/H5N1 clade) and NA (where the sequence is placed as a sister to a H3N2 sequence) (Additional file 1: Figure S1). Also, the network diagram indicates this SPR modification for the HA and NA trees (blue edge, Figure 5). This scenario indicates one or more reassortment events where hk1073 is formed by acquiring gene segments encoding internal proteins from a H6N1 virus, or one with the same composition of genes. It was reported previously that this H9N2 isolate shares the same six internal segments with H5N1 and H9N2 strains circulating in 1997 [40], as well as with H6N1 1997 strains [41]. Another significant SPR modification involves moving sh39 to cluster with the quail isolates (quail99) of type H6N1 (Figure 6C), suggested by the HA tree only. This grouping is altered in the internal segments’ trees by the presence of the hk1073 isolate, resulting in sh39 being an outgroup of the quail99/hk1073 clade (MP) or a sister clade to hk1073 (NP, NS, PB1, PB2). The result reinforces the hypothesis that the hk1073 is a reassortant in the H6N1 group.

The proposed scenario is that hk1073 is a reassortant virus that is made up of internal gene segments derived from H6N1 viruses (sh39 and quail isolates), an NA from a H3N2-like virus, and a HA from another H9 virus. The segments resulting in this genome architecture could have been incorporated into this virus at the same time in one reassortment event, or this could have occurred in many stages. A possibility is that hk1073 first acquired an NA segment from an HxN2 strain (Hx stands for any type of HA), internal segments from an H6N1-like virus that circulated in 1999 and a HA segment from either parent, generating an intermediate type. This newly formed strain would be involved in another event that gave rise to the hk1073 reassortant by acquiring a HA segment from an H9Nx virus (where Nx stands for any type of NA). Previous work [41] suggests that the H9N2 viruses isolated in Hong Kong in 1997 shared the segments encoding for internal proteins with the H6N1 and H5N1 viruses of that year. Also, Lin and colleagues [42] report that the hk1073 strain itself has not picked up any segments by reassortment since 1997 and that it is closely related to A/quail/HongKong/G1/1997. This leads us to believe that the 1997 precursor of hk1073 is the first reassortant strain with this genetic make up from which the 1999 isolates were generated. Influenza H9N2 strains in general have been observed to possess a high propensity for reassortment [43].

It is expected that the outgroup strain would not be involved in any reassortment events (in this analysis, the outgroup was isolated on a different continent ten years previously). However, there are some SPR operations that incorporate this sequence, which could point to reassortment events with other strains that were not included in our dataset. None of the branch swaps involving the outgroup strain appear in the results from both algorithms, therefore we didn’t consider them. Another SPR (grouping A/Environment/HongKong/43710/99 H5N1 or env99, with hk1774) was found by both algorithms (Figure 6D), but there is little agreement on which segments propose this move. The HA tree disagrees with the PB2 tree on the grouping of the hk1774 and env99 strains. It seems that these two gene segments have evolved separately, however there is not enough information to hypothesise how this may have happened and what is the source of the other segments.

In the reassortment network, the PA node is not connected by edges to any other nodes (Figure 5). Poorly supported branches in the phylogenetic topology can cause this effect. Another challenge is to determine which SPR operations are complementary to each other and this can only be done in the context of all the other trees. One of the limitations with EEEP is memory consumption [25] but most paths in pairwise comparisons were recovered. The main drawback in using an individual phylogeny as a starting point for the algorithm is the potential of wrongly inferring reassortment events if the start topology is incorrect. Comparing Bayesian phylogenetic tree sets in geometrical space is one way of approaching the comparison of large sets of trees. MDS was found useful in comparing multiple Bayesian analyses and for exploring sets of trees [44]. However, its challenges include information loss during the scaling of distances into lower dimensions of space and defining tree space from a limited number of trees. Another approach could consist of constraining the MCMC tree search by using another segment’s topology, and using Bayes factors [45] to determine whether one hypothesis describes the data significantly better than the other. Nevertheless, the algorithms presented here have the potential to detect real reassortment events by minimising the inference of false positives.

## Conclusions

Two statistical frameworks for identifying reassortment events using phylogeny are presented. The first algorithm (*MLreassort*) uses a maximum likelihood approach. Here we identify the shortest path (consisting of SPR operations) for converting a phylogenetic tree derived from gene A to the tree derived from gene B. An SPR operation is evaluated as significant when the resulting tree has a significantly lower likelihood score compared to the pre-modified tree. This was carried out for all the pairwise comparisons of trees and interpretation of results is done considering all the trees and the proposed SPR modifications. The second algorithm is based on a Bayesian approach (*Breassort*). The steps consist of identifying non-overlapping tree spaces, detecting SPR moves that would convert trees from the tree space of gene A to trees from that of gene B (200 comparisons), and focusing on the most frequent branch swaps. The SPR operations that are considered to result in significant changes in tree topology are events that cannot be simply explained by errors in the phylogenetic estimation and, having rejected the hypothesis of recombination, reassortment needs to be considered. We use a conservative approach when inferring reassortment events by only considering those SPRs that are found by both algorithms. Further work will involve development of the algorithm for use on larger data sets. As the size of the data set increases, it is likely that more reassortant strains would be included and a better way of representing the results would also be necessary. Nevertheless, the algorithms presented are able to detect reassortment events in any segmented viruses, using statistical methods for distinguishing between true reassortment and other causes of incongruence between phylogenetic tree topologies.

Automated computational methods for detecting reassortment events have been explored previously and these involve or avoid the use of phylogenetic analysis. Suzuki’s phylogenetic approach [46] is based on examining quartets of strains; the FluReF [47] algorithm selects candidate reassortment groups through a bottom-up search of phylogenetic trees, and another approach combines genotyping and phylogenetics to infer reassortment events [48]. Recently, Nagarajan and Kingsford [49] reported GiRaF, a graph-incompatibility-based reassortment finder. The program involves the generation of an incompatibility graph from sets of trees resulting from a Bayesian tree search, followed by mining of phylogenetic discordances using a search algorithm. In contrast with our algorithm, GiRaF can be used to process large datasets efficiently. In addition to the detection of reassortment events, GiRaF also reports a measure of confidence for each prediction. Rabadan et al. have developed a statistical method to estimate the likelihood that two segments have co-evolved, without relying on phylogenetic analysis [50]. Another algorithm based on the theory of quasispecies was reported by Wan et al. [51], where using segment genotype thresholds allowed the identification of reassortment events. Macken and colleagues [52] established a baseline of circulating genotypes at a certain time period, and used it to characterize reassortment events of viruses from a second time period. Another algorithm based on neighbourhood of strains for each segment [53] was developed to detect reassortment in all the unique full genomes available until June 2011. MDS has also been used to identify differences in influenza A phylogenetic history. Rambaut et al. [54] used MDS to plot the distances between 500 trees for segments derived from H3N2 isolates. In this case, the tree distances were calculated from the time to the most recent common ancestor for each season. Although some of these methods are more efficient in processing large datasets, we believe that our approaches provide a sensitive and accurate test for the detection of reassortment events.

## Methods

### Data selection

We used the Influenza Virus Resource at NCBI [55] to extract the nine sequences of the fully completed genomes isolated in Hong Kong during 1999, using as outgroup A/redknot/NJ/325/1989 H7N7 (accession numbers provided in Additional file 1: Table S1). This set includes four strains isolated from the environment. We kept only one of these sequences due to their high similarity (≥99%). The dataset then consisted of one H5N1 virus isolated from the environment, three H6N1 strains from quail and pheasant, one human H9N2, one human H3N2 strain and a H7N7 isolate as the outgroup. Data deposited in the Dryad Repository: http://dx.doi.org/10.5061/dryad.mg040.

### Alignments and test for recombination

We compiled alignments for each of the 8 segments using MUSCLE [56] with the parameters set to their default values. The alignments were inspected by eye to see if any ambiguously aligned regions were included. The alignments were examined using the PTP (permutation probability) test implemented in PAUP [57] and also analysed with likelihood mapping implemented in TreePuzzle [58] (default parameters used in both cases). The best fitting model of nucleotide substitution was selected by evaluating 56 compositionally homogenous models using the Akaike information criterion test implemented in the ModelTest software [59]. In order to eliminate the possibility that variation in phylogenetic tree topologies is due to the presence of recombinant segments, the PHI (Pairwise Homoplasy Index) test [60] for recombination was applied to each of the eight alignments, with the default parameters. The program uses the alignment in question and returns files with informative and unambiguous sites as well as a log file containing the PHI p-values.

### Phylogenetic analysis

PAUP*4b10 [57] was used to carry out the maximum likelihood calculations. When the best fitting models were identified, PAUP was used in order to find the maximum likelihood tree topology. We used the Tree-Bisection-Reconnection method with 10 random addition replicates. The software program MrBayes v3.1.2 [61] was used to carry out the Bayesian analysis. The program was run twice for each segment with 4 chains and sampling frequency of 1000, for 10 million generations with 10% of the sampled trees discarded as ‘burn-in’. We checked for convergence of the two runs by using the standard deviation of split frequencies, which is a measure of the similarity of the tree samples of the two independent runs. As the two tree samples become increasingly similar, the average standard deviation of split frequencies approaches zero. Only the trees sampled after this convergence point were used for further analysis.

### Tree distance measures

In order to get a general overview of the differences between tree topologies, Robinson-Foulds (RF) distances between the trees were calculated using PAUP. We then carried out 56 pairwise comparisons (all possible pairwise comparisons of the 8 segments) using the EEEP (Efficient Evaluation of Edit Paths) software [25]. EEEP is a program that seeks to reconcile a reference tree and a test tree, by finding the minimum number of SPR moves required to convert the former into the latter. The output of the program consists of the smallest set of SPR permutations that could convert the reference tree topology into the test tree topology. Such a set of SPR operations is called an edit path, and it is possible for more than one path to be found. The goal is to find the shortest edit path, or the smallest number of SPR operations that can reconcile the reference and test trees. As these transfers are analogous to lateral gene transfer (LGT) events, the edit path output by the program corresponds to a minimum set of lateral gene transfers between strains in the reference tree. The following settings were used: partitioning of data into regions of discordance, using time constraints and permissive tree distance ratchets (see original publication for details of the algorithm). Ratchets can be used to assess whether a modified reference tree is more or less similar to the test tree than the pre-modification tree. The use of ratchets can reduce the running time of the algorithm but the frequency of the cases where no solution is found increases [25]. EEEP was run using a reference tree, a test tree and a support threshold of 0, which will not collapse any nodes into polytomies. Perl scripts were developed to run the software on each of the eight segments’ rooted trees using each of the other seven segments’ unrooted trees as test trees, and to parse the output. The SPR moves suggested by EEEP were used to modify the reference tree (code available on request) and a set of intermediate trees was compiled for each pairwise comparison.

### AU test

In order to run the AU test, PAUP was used to produce a text file with the site-wise log-likelihoods for each set of trees resulting from the EEEP analysis. This log-likelihood file was used as input for CONSEL [62]. CONSEL is a program package for assessing the confidence of tree selection i.e. to evaluate which trees are within the confidence set. The confidence set, the set of trees that are not rejected by the tests, is expected to include the true tree [28]. The output consists of a list of p-values for each of the trees in the input file, one of which corresponds to the AU test statistic. We used a p-value cut-off of 0.01 to determine the confidence sets. For all the trees in the confidence, we can suggest that the differences between them are not statistically significant and can be explained by sampling artifacts. Trees outside the confidence set are a significantly worse explanation of the data. The null hypothesis (that there is no reassortment) is rejected by the trees with a p-value lower than a chosen threshold. The approximately unbiased (AU) test (implemented in CONSEL) was run twice in order to obtain confidence intervals around both the reference and test trees. To find the confidence interval around a reference tree, the site-wise log likelihoods were calculated using the alignment for the reference tree. To find the confidence interval around the test tree, the procedure was repeated but this time the alignment for the test tree was used when getting the log likelihoods. (Additional file 1: Figure S2) shows diagrammatically how an SPR is determined to be significant, given different SPR paths between two confidence sets of trees. The interpretation is as follows.

A significant branch move (or edit) is one that, when applied to a tree, causes a significant change in tree topology. Additional file 1: Figure S2a and Sb shows those instances where the trees on the edit path are always within the confidence set of optimal trees of at least one alignment. In these cases, by definition, there are no significant transitions. In the first case, the two confidence intervals overlap and in the second, the confidence set for Tr (reference tree) is a subset of the confidence set for Tt (test tree). In Additional file 1: Figure S2c and Sd, the edit path connects two non-overlapping confidence sets. In both cases, we would consider that LGT is the best explanation of the observed data. If multiple transfers are found in each path between these confidence intervals, the shortest path is considered (Additional file 1: Figure S2e). If transitions in a path move in and out of a confidence interval, the last transition that moves out of the confidence region is considered (Additional file 1: Figure S2f). When multiple trees are found outside both confidence sets, pairwise analysis of neighbouring trees needs to be carried out again using the AU test, in order to determine the significance of the transitions that connect them.

### Representing trees in space

Multidimensional scaling, also known as principle coordinate analysis, is a way of representing pairwise distances between objects [32]. The idea is based on ordering points (representing phylogenetic trees) in geometrical space such that similar points are closer to each other. Pairwise tree sets were compared by visually representing them in 2D space. In order to do this, we combined the sets of trees for two segments at a time (26 pairwise sets) and obtained the Robinson-Foulds distance between all trees in the combined sets (this includes distances between trees within a set, and between the two sets). The resulting matrix was analysed using the R statistical software [63]. The high dimensionality of the data makes it impossible to explain and visualize the observed distances between the trees and therefore multi-dimensional scaling was used to reduce the data to two dimensions. The stress, which is a measure of the squared differences between ideal distances and actual distances, needs to be minimized so as to better reflect the original distances between objects. A standard statistical method for ordering points into convex hulls was applied [33]. Convex hull peeling was carried out to remove the outermost points in a cloud (e.g. 5%) in order to eliminate outliers. The remaining 95% hulls were compared. A plot of two sets of points was obtained, representing trees derived from two different segments. If the clouds of points (more precisely, their 95% confidence sets) overlap, we conclude that no significant differences between the tree topologies in the two sets exist. However, if there is no overlap, we can hypothesize that the trees in the two sets reflect different evolutionary histories. In this case we carried out EEEP analysis between the two clouds of trees. We carried out 200 EEEP analyses (100 in one direction, and 100 in reverse) between the two sets, randomly picking the trees to compare. This resulted in a set of significant SPR branch moves. We deemed any SPR modification that was present in more than 70% of paths between two tree sets as worthy of consideration. Python packages reportlab and rpygraphviz were used to draw a frequency (or reassortment) network to visually represent these results.

### Combining results from both approaches

Plots were used to visualize the overlap between the results from the maximum likelihood and Bayesian approaches together. The lists of SPR moves from each algorithm were compared and a plot was drawn for each SPR found by both approaches. The *x*-axis shows the eight segments and any modifications their corresponding trees (or tree sets) propose. The *y*-axis shows the trees that need to be modified. For example, if the HA tree proposes an SPR move (say hk1073 to hk1774, Figure 6A) to the MP tree, this will be illustrated by a mark at the HA *x*-axis and MP *y-*axis positions. The marks can be either circles or crosses. A circle indicates that the SPR comes from the *MLreassort*, whereas a cross indicates a result from the *Breassort*. We took this conservative approach order to eliminate method-dependent inferences of reassortment.

### Simulated data

The algorithms were tested on a simulated dataset in order to assess their capacity to detect manually induced reassortment events. The program Seq-Gen [64] was used to simulate eight alignments on a tree derived from the HA segment of the Hong Kong data set. An alignment of seven sequences was generated for each gene segment using the GTR model of nucleotide substitution, and the alignment length is equivalent to the length of the respective gene (e.g. the simulated alignment for HA is 1,778 bp in length). The sequences were named using letters A-G. Phylogenetic trees were built from these alignments and were rooted on taxon ‘A’. An SPR modification was then manually introduced in the tree used to generate the HA data, by moving taxon ‘G’ to group with taxon ‘B’ rather than with taxon ‘F’. The algorithms were reapplied to the simulated data with the modified HA alignment, in order to test whether they can detect this manually induced SPR.

## Competing interests

The authors declare no competing interests.

## Authors’ contributions

VS carried out the work under the supervision of JOMcI. JOMcI conceived the original idea, participated in its design and coordination and helped to draft the manuscript. JAC contributed with useful ideas during the initial development and with support for the statistical aspects of the work. All authors read and approved the final manuscript.

## Supplementary Material

Additional file 1 **Figure S1.** Maximum likelihood trees with significant branch swaps. **Figure S2** – Possible relationships between two confidence sets. **Figure S3** – Effect of varying confidence intervals thresholds on reassortment networks. **Table S1:** Accession numbers of sequences used in this study. Click here for file

## References

[B1] Webster RG Bean WJ Gorman OT Chambers TM Kawaoka Y Evolution and ecology of influenza A viruses Microbiol Mol Biol Rev 1992 56 1 152 179 10.1128/mr.56.1.152-179.1992 PMC372859 1579108

[B2] Bouvier NM Palese P The biology of influenza viruses Vaccine 2008 26 Suppl 4 D49 D53 19230160 10.1016/j.vaccine.2008.07.039 PMC3074182

[B3] Lamb RA Choppin PW The gene structure and replication of influenza virus Annu Rev Biochem 1983 52 1 467 10.1146/annurev.bi.52.070183.002343 6351727

[B4] Kilbourne ED Future influenza vaccines and the use of genetic recombinants Bull World Health Organ 1969 41 3 643 645 5309489 PMC2427719

[B5] Laver WG Webster RG Studies on the origin of pandemic influenza. II. Peptide maps of the light and heavy polypeptide chains from the hemagglutinin subunits of A 2 influenza viruses isolated before and after the appearance of Hong Kong influenza Virology 1972 48 2 445 455 10.1016/0042-6822(72)90055-4 5024608

[B6] Laver WG Webster RG Studies on the origin of pandemic influenza. 3. Evidence implicating duck and equine influenza viruses as possible progenitors of the Hong Kong strain of human influenza Virology 1973 51 2 383 391 10.1016/0042-6822(73)90437-6 4632653

[B7] Hilleman MR Realities and enigmas of human viral influenza: pathogenesis, epidemiology and control Vaccine 2002 20 25–26 3068 3087 12163258 10.1016/s0264-410x(02)00254-2

[B8] Smith GJ Vijaykrishna D Bahl J Lycett SJ Worobey M Pybus OG Ma SK Cheung CL Raghwani J Bhatt S Origins and evolutionary genomics of the 2009 swine-origin H1N1 influenza A epidemic Nature 2009 459 7250 1122 1125 10.1038/nature08182 19516283

[B9] Forrest HL Webster RG Perspectives on influenza evolution and the role of research Anim Health Res Rev 2010 11 1 3 18 10.1017/S1466252310000071 20591210

[B10] Li C Hatta M Nidom CA Muramoto Y Watanabe S Neumann G Kawaoka Y Reassortment between avian H5N1 and human H3N2 influenza viruses creates hybrid viruses with substantial virulence Proc Natl Acad Sci USA 2010 107 4687 4692 10.1073/pnas.0912807107 20176961 PMC2842136

[B11] Felsenstein J Inferring phylogenies 2004 Sunderland, MA: Sinauer Associates

[B12] Nelson MI Holmes EC The evolution of epidemic influenza Nat Genet 2007 8 196 205 10.1038/nrg2053 17262054

[B13] Chen R Holmes EC The evolutionary dynamics of human influenza B virus J Mol Evol 2008 66 655 663 10.1007/s00239-008-9119-z 18504518 PMC3326418

[B14] Guan Y Peiris M Kong KF Dyrting KC Ellis TM Sit T Zhang LJ Shortridge KF H5N1 influenza viruses isolated from geese in southeastern China: Evidence for genetic reassortment and interspecies transmission to ducks Virology 2002 292 16 23 10.1006/viro.2001.1207 11878904

[B15] Holmes EC Ghedin E Miller N Taylor J Bao Y St George K Grenfell BT Salzberg SL Fraser CM Lipman DJ Taubenberger JK Whole-genome analysis of human influenza a virus reveals multiple persistent lineages and reassortment among recent H3N2 viruses PLoS Biol 2005 3 9 1579 1589 10.1371/journal.pbio.0030300 PMC1180517 16026181

[B16] Nelson MI Viboud C Simonsen L Bennett RT Griesemer SB St George K Taylor J Spiro DJ Sengamalay NA Ghedin E Taubenberger JK Holmes EC Multiple reassortment events in the evolutionary history of H1N1 Influenza A virus since 1918 PLoS Pathog 2008 4 2 e1000012 10.1371/journal.ppat.1000012 18463694 PMC2262849

[B17] Keane TM Creevey CJ Pentony MM Naughton TJ Mclnerney JO Assessment of methods for amino acid matrix selection and their use on empirical data shows that ad hoc assumptions for choice of matrix are not justified BMC Evol Biol 2006 6 29 10.1186/1471-2148-6-29 16563161 PMC1435933

[B18] Smith VS Page RDM Johnson KP Data incongruence and the problem of avian louse phylogeny Zoologica Scripta 2004 33 3 239 259 10.1111/j.0300-3256.2004.00149.x

[B19] Bergsten J A review of long-branch attraction Cladistics 2005 21 163 193 10.1111/j.1096-0031.2005.00059.x 34892859

[B20] Graybeal A Is it better to add taxa or characters to a difficult phylogenetic problem? Syst Biol 1998 47 9 17 10.1080/106351598260996 12064243

[B21] Chippindale PT Wiens JJ Weighting, partitioning, and combining characters in phylogenetic analysis Syst Biol 1994 43 2 278 287

[B22] Robinson DF Foulds LR Comparison of phylogenetic trees Math Biosci 1981 53 1–2 147

[B23] Robinson DF Comparison of labelled trees with valency three J Combinatorial Theory, Series B 1971 11 2 105 119 10.1016/0095-8956(71)90020-7

[B24] Allen BL Steel M Subtree transfer operations and their induced metrics on evolutionary trees Annals of Combinatorics 2001 5 1 1 15 10.1007/s00026-001-8006-8

[B25] Beiko RG Hamilton N Phylogenetic identification of lateral genetic transfer events BMC Evol Biol 2006 6 15 10.1186/1471-2148-6-15 16472400 PMC1431587

[B26] Felsenstein J Confidence limits on phylogenies: an approach using the bootstrap Evolution 1985 39 783 791 10.2307/2408678 28561359

[B27] Kishino H Hasegawa M Evaluation of the maximum likelihood estimate of the evolutionary tree topologies from DNA sequence data, and the branching order in hominoidea J Mol Evol 1989 29 2 170 179 10.1007/BF02100115 2509717

[B28] Shimodaira H An approximately unbiased test of phylogenetic tree selection Syst Biol 2002 51 3 492 508 10.1080/10635150290069913 12079646

[B29] Haggerty LS Martin FJ Fitzpatrick DA McInerney JO Gene and genome trees conflict at many levels Philos Trans R Soc Lond B 2009 364 2209 2219 10.1098/rstb.2009.0042 19571241 PMC2873008

[B30] Magiorkinis G Ntziora F Paraskevis D Magiorkinis E Hatzakis A Analysing the evolutionary history of HCV: Puzzle of ancient phylogenetic discordance Infect Genet Evol 354 7 3 354 16720108 10.1016/j.meegid.2006.04.003

[B31] Poptsova M Gogarten P The power of phylogenetic approaches to detect horizontally transferred genes BMC Evol Biol 2007 7 1 45 10.1186/1471-2148-7-45 17376230 PMC1847511

[B32] Rabinowitz GB An introduction to nonmetric multidimensional scaling Am J Pol Sci 1975 19 2 343 390 10.2307/2110441

[B33] Barnett V The ordering of multivariate data J R Stat Soc Series A 1976 139 3 318 10.2307/2344839

[B34] Faith DP Cranston PS Could a cladogram this short have arisen by chance alone? On permutation tests for cladistic structure Cladistics 1991 7 1 1 28 10.1111/j.1096-0031.1991.tb00020.x

[B35] Strimmer K von Haeseler A Likelihood-mapping: a simple method to visualize phylogenetic content of a sequence alignment Proc Natl Acad Sci 1997 94 13 6815 6819 10.1073/pnas.94.13.6815 9192648 PMC21241

[B36] Foster PG Modeling compositional heterogeneity Syst Biol 2004 53 485 495 10.1080/10635150490445779 15503675

[B37] Benjamini Y Hochberg Y Controlling the false discovery rate - a practical and powerful approach to multiple testing J Roy Stat Soc B Met 1995 57 1 289 300

[B38] Rodriguez F Oliver JL Marin A Medina JR The general stochastic model of nucleotide substitution J Theor Biol 1990 142 4 485 501 10.1016/S0022-5193(05)80104-3 2338834

[B39] Posada D Crandall KA Selecting models of nucleotide substitution: an application to human immunodeficiency virus 1 (HIV-1) Mol Biol Evol 2001 18 6 897 906 10.1093/oxfordjournals.molbev.a003890 11371577

[B40] Lin YP Shaw M Gregory V Cameron K Lim W Klimov A Subbarao K Guan Y Krauss S Shortridge K Avian-to-human transmission of H9N2 subtype influenza A viruses: relationship between H9N2 and H5N1 human isolates Proc Natl Acad Sci U S A 2000 97 17 9654 9658 10.1073/pnas.160270697 10920197 PMC16920

[B41] Hoffmann E Stech J Leneva I Krauss S Scholtissek C Chin PS Peiris M Shortridge KF Webster RG Characterization of the influenza A virus gene pool in avian species in southern China: was H6N1 a derivative or a precursor of H5N1? J Virol 2000 74 14 6309 6315 10.1128/JVI.74.14.6309-6315.2000 10864640 PMC112136

[B42] Lin YP Shaw M Gregory V Cameron K Lim W Klimov A Subbarao K Guan Y Krauss S Shortridge K Avian-to-human transmission of H9N2 subtype influenza A viruses: relationship between H9N2 and H5N1 human isolates Proc Natl Acad Sci 2000 97 17 9654 9658 10.1073/pnas.160270697 10920197 PMC16920

[B43] Xu KM Li KS Smith GJ Li JW Tai H Zhang JX Webster RG Peiris JS Chen H Guan Y Evolution and molecular epidemiology of H9N2 influenza A viruses from quail in southern China, 2000 to 2005 J Virol 2007 81 6 2635 2645 10.1128/JVI.02316-06 17192315 PMC1865985

[B44] Hillis DM Heath TA St John K Analysis and visualization of tree space Syst Biol 2005 54 3 471 482 10.1080/10635150590946961 16012112

[B45] Kass RE Raftery AE Bayes factors J Am Stat Assoc 1995 90 430 773 10.1080/01621459.1995.10476572

[B46] Suzuki Y A phylogenetic approach to detecting reassortments in viruses with segmented genomes Gene 2010 464 1–2 11 16 20546849 10.1016/j.gene.2010.05.002

[B47] Yurovsky A Moret BME FluReF, an automated flu virus reassortment finder based on phylogenetic trees BMC Genomics 2011 12 Suppl 2 S3 10.1186/1471-2164-12-S2-S3 21989112 PMC3194234

[B48] Dong CZ Ying LY Yuan DF Detecting transmission and reassortment events for influenza A viruses with genotype profile method Virol J 2011 8 395 10.1186/1743-422X-8-395 21824442 PMC3162547

[B49] Nagarajan N Kingsford C GiRaF: robust, computational identification of influenza reassortments via graph mining Nucleic Acids Res 2011 39 6 e34 10.1093/nar/gkq1232 21177643 PMC3064795

[B50] Rabadan R Levine AJ Krasnitz M Non-random reassortment in human influenza A viruses Influenza Other Respi Viruses 2008 2 1 9 22 10.1111/j.1750-2659.2007.00030.x 19453489 PMC4634327

[B51] Wan XF Chen G Luo F Emch M Donis R A quantitative genotype algorithm reflecting H5N1 Avian influenza niches Bioinformatics 2007 23 18 2368 2375 10.1093/bioinformatics/btm354 17623701

[B52] Macken CA Webby RJ Bruno WJ Genotype turnover by reassortment of replication complex genes from avian influenza A virus J Gen Virol 2006 87 Pt 10 2803 2815 16963738 10.1099/vir.0.81454-0

[B53] de Silva UC Tanaka H Nakamura S Goto N Yasunaga T A comprehensive analysis of reassortment in influenza A virus Biology Open 2012 1 4 385 390 10.1242/bio.2012281 23213428 PMC3509451

[B54] Rambaut A Pybus OG Nelson MI Viboud C Taubenberger JK Holmes EC The genomic and epidemiological dynamics of human influenza A virus Nature 2008 453 7195 615 619 10.1038/nature06945 18418375 PMC2441973

[B55] Bao Y Bolotov P Dernovoy D Kiryutin B Zaslavsky L Tatusova T Ostell J Lipman D The influenza virus resource at the national center for biotechnology information J Virol 2008 82 2 596 601 10.1128/JVI.02005-07 17942553 PMC2224563

[B56] Edgar RC MUSCLE: multiple sequence alignment with high accuracy and high throughput Nucleic Acid Res 2004 32 5 1792 1797 10.1093/nar/gkh340 15034147 PMC390337

[B57] Swofford DL PAUP*: phylogenetic analysis using parsimony (*and other methods), version 4.0b10 1998 Sunderland: Sinauer Associates

[B58] Schmidt HA Strimmer K Vingron M von Haeseler A Tree-puzzle: maximum likelihood phylogenetic analysis using quartets and parallel computing Bioinformatics 2002 18 3 502 504 10.1093/bioinformatics/18.3.502 11934758

[B59] Posada D Crandall KA MODELTEST: testing the model of DNA substitution Bioinformatics 1998 14 9 817 818 10.1093/bioinformatics/14.9.817 9918953

[B60] Bruen TC Philippe H Bryant D A simple and robust statistical test for detecting the presence of recombination Genetics 2006 172 4 2665 2681 16489234 10.1534/genetics.105.048975 PMC1456386

[B61] Ronquist F Huelsenbeck JP MrBayes 3: Bayesian phylogenetic inference under mixed models Bioinformatics 2003 19 12 1572 1574 10.1093/bioinformatics/btg180 12912839

[B62] Shimodaira H Hasegawa M CONSEL: for assessing the confidence of phylogenetic tree selection Bioinformatics 2001 17 12 1246 1247 10.1093/bioinformatics/17.12.1246 11751242

[B63] R Development Core Team R: a language and environment for statistical computing 2009 Vienna, Austria: R Foundation for Statistical Computing

[B64] Rambaut A Grassly NC Seq-Gen: an application for the monte carlo simulation of DNA sequence evolution along phylogenetic trees Comput Appl Biosci 1997 13 3 235 238 9183526 10.1093/bioinformatics/13.3.235

