# Supplementary material for: New approaches for unravelling reassortment pathways
Source: BMC Evol Biol. 2013 Jan 1;13:1. doi: 10.1186/1471-2148-13-1 (PMC3541980; doi:10.1186/1471-2148-13-1)
Supplement: Additional file 1 — Figure S1. Maximum likelihood trees with significant branch swaps. Figure S2 – Possible relationships between two confidence sets. Figure S3 – Effect of varying confidence intervals thresholds on reassortment networks. Table S1: Accession numbers of sequences used in this study. [file 1471-2148-13-1-S1.pdf]

Supplementary Figures and Tables

Figure S1. Maximum likelihood trees with significant branch swaps

Arcs point from the branch that needs to be moved, to the recipient branch. Direction is from empty circle to black-filled circle. Bidirectional arcs refer to transfers that can occur in both directions. Legend indicates which segments propose a particular move, identified by colours. Bootstrap values are shown above nodes. Each arc corresponds to an SPR move.

Figure S2. Possible relationships between two confidence sets

Red arrows symbolise significant transitions, while black arrows are insignificant ones; Tr – reference tree; Tt – test tree; t1…tn - tree numbers in the order generated in the previous step. Green and pink ovals symbolize the confidence sets of the reference and test trees respectively. (a), (b) Confidence sets overlap; all transitions are inside either confidence set - no significant ones. (c) Transition that connects trees from two non- overlapping confidence sets is significant. (d) There can be a number of moves connecting the two confidence intervals. In the case where there is more than one tree outside both confidence intervals, separate analysis on these has been applied (using the AU test) to assess whether there is significant difference between them. (e) Multiple paths could be found connecting non-overlapping confidence sets. If the paths are of varying lengths, the shortest ones are considered (f) Only the last transition, that connects a tree from within the confidence set of the reference tree to a tree outside both sets, is taken into account. Similarly, the first transition that connects a tree outside both sets to one within the confidence set of the test tree is considered.

Figure S3. Effect of varying confidence intervals thresholds on reassortment networks

Different thresholds (90%, 95%, 99%, 99.9%) for determining confidence intervals (CIs) were applied, and analysis repeated three times in each case. The reference network is the one presented in the report. Dashed lines indicate SPR moves not found in the reference network, while faint crossed lines (X) represent transitions that are present in the reference but missing in that particular network. The variations observed between the different CIs are not greater than those between networks generated using the same CI parameters.

Table S1. Accession numbers of sequences used in this study

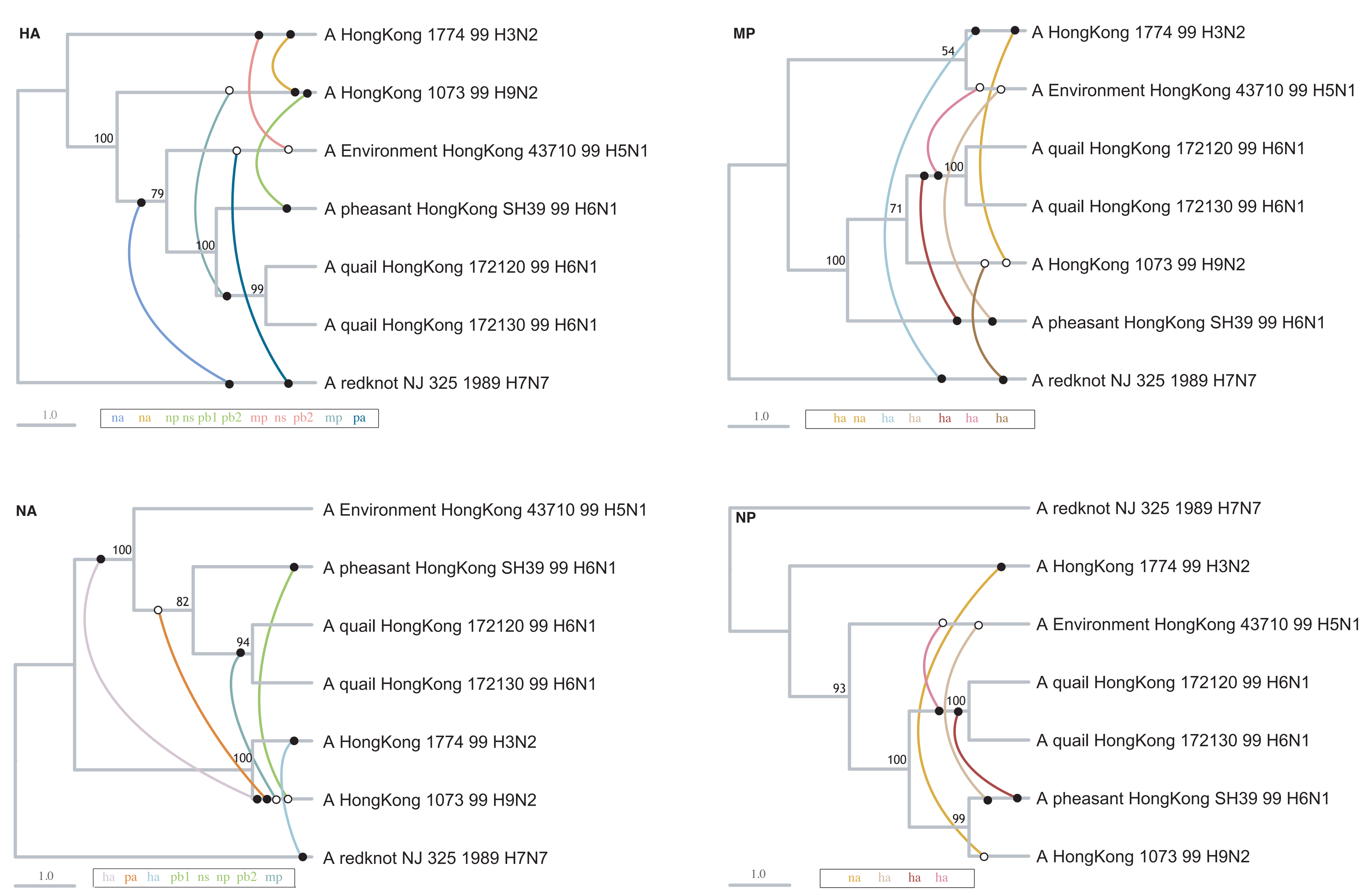

HA
A HongKong 1774 99 H3N2
A HongKong 1073 99 H9N2
A Environment HongKong 43710 99 H5N1
A pheasant HongKong SH39 99 H6N1
A quail HongKong 172120 99 H6N1
A quail HongKong 172130 99 H6N1
A redknot NJ 325 1989 H7N7
100
79
100
99
1.0
na na np ns pb1 pb2 mp ns pb2 mp pa
MP
A HongKong 1774 99 H3N2
A Environment HongKong 43710 99 H5N1
A quail HongKong 172120 99 H6N1
A quail HongKong 172130 99 H6N1
A HongKong 1073 99 H9N2
A pheasant HongKong SH39 99 H6N1
A redknot NJ 325 1989 H7N7
54
100
71
100
1.0
ha na ha ha ha ha ha
NA
A Environment HongKong 43710 99 H5N1
A pheasant HongKong SH39 99 H6N1
A quail HongKong 172120 99 H6N1
A quail HongKong 172130 99 H6N1
A HongKong 1774 99 H3N2
A HongKong 1073 99 H9N2
A redknot NJ 325 1989 H7N7
100
82
94
100
1.0
ha pa ha pb1 ns np pb2 mp
NP
A redknot NJ 325 1989 H7N7
A HongKong 1774 99 H3N2
A Environment HongKong 43710 99 H5N1
A quail HongKong 172120 99 H6N1
A quail HongKong 172130 99 H6N1
A pheasant HongKong SH39 99 H6N1
A HongKong 1073 99 H9N2
93
100
100
99
1.0
na ha ha ha

Figure S1

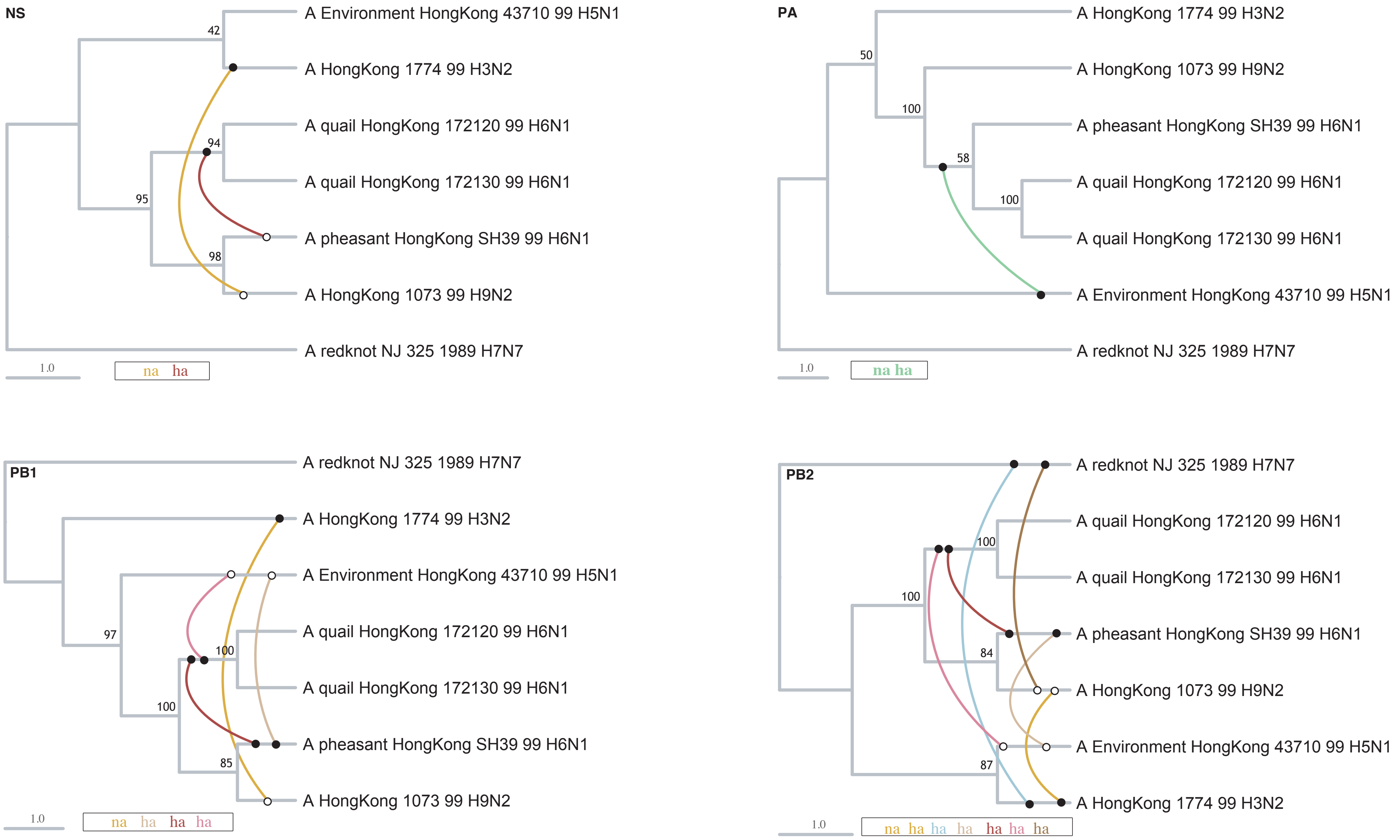

Figure S1 (continued)

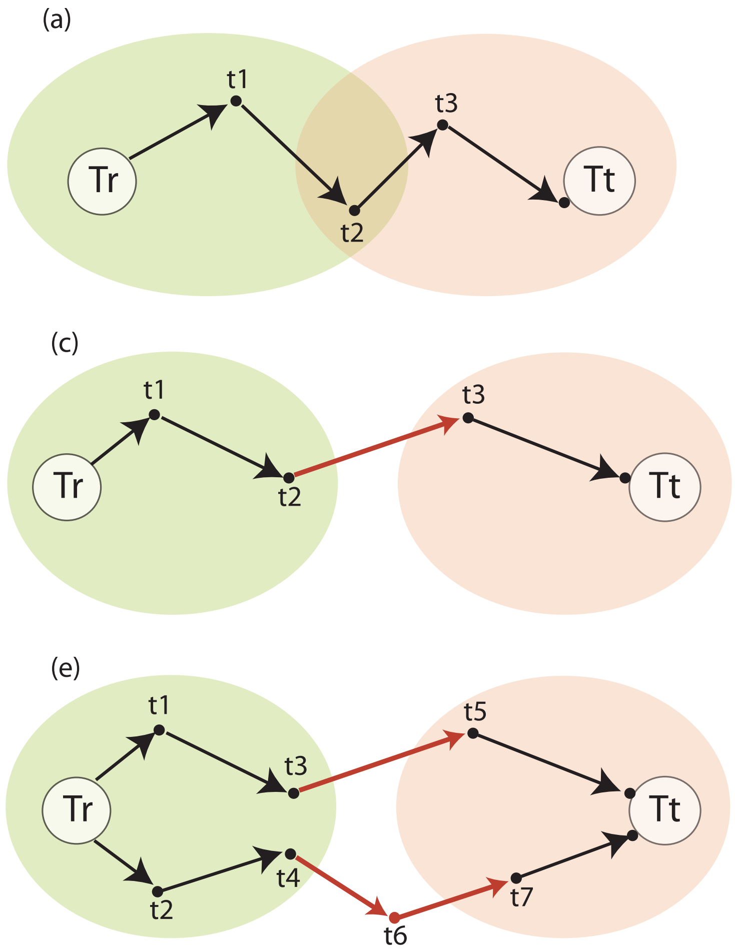
(a)
t1
t3
Tr
Tt
t2
(b)
t1
t3
Tr
t2
Tt
(c)
t1
t3
Tr
Tt
t2
(d)
t1
t3
Tr
Tt
t2
(e)
t1
t5
t3
Tr
Tt
t4
t2
t7
t6
(f)
t1
t2
t3
t5
Tr
Tt
t4

Figure S2

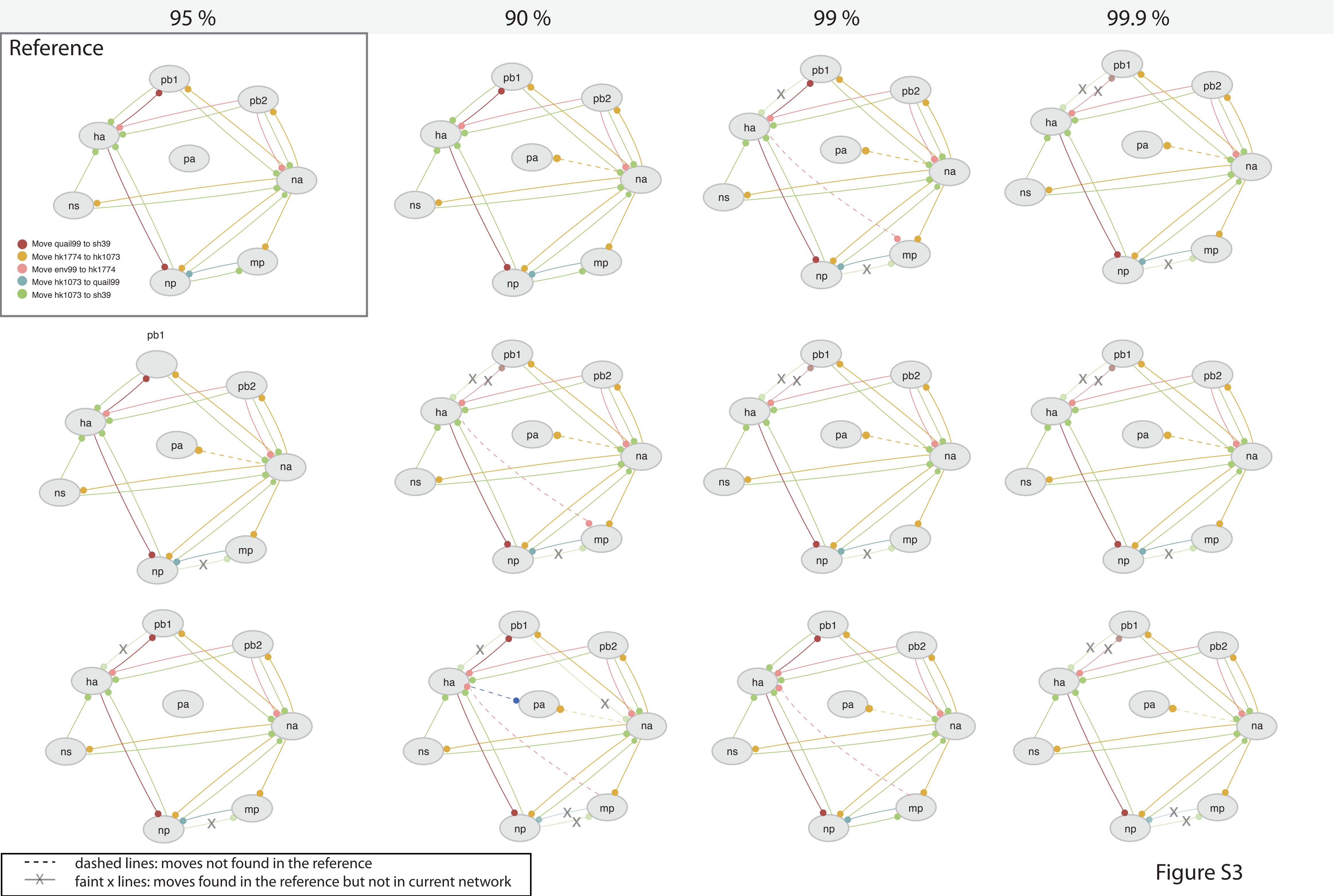
95 %
90 %
99 %
99.9 %
Reference
pb1
pb2
ha
pa
na
ns
mp
np
Move quail99 to sh39
Move hk1774 to hk1073
Move env99 to hk1774
Move hk1073 to quail99
Move hk1073 to sh39
dashed lines: moves not found in the reference
faint x lines: moves found in the reference but not in current network

Figure S3

Table S1. Accession numbers of sequences used in this study.

Strain	Accession numbers
A/redknot/NJ/325/1989 H7N7	CY005079 - CY005085, CY005981
A/environment/HongKong/43710/1999 H5N1	AF216734 - AF216741
A/pheasant/HongKong/SH39/1999 H6N1	AJ410495, AJ410502, AJ410511, AJ410519, AJ410548, AJ410557, AJ410568, AJ410577
A/quail/HongKong/172120/1999 H6N1	AJ410496, AJ410503, AJ410512, AJ410520, AJ410549, AJ410558, AJ410569, AJ410578
A/quail/HongKong/172130/1999 H6N1	AJ410497, AJ410504, AJ410513, AJ410521, AJ410550, AJ410559, AJ410570, AJ410579
A/HongKong/1774/1999 H3N2	CAC40037 – CAC40044, CAC40062 - CAC40063
A/HongKong/1073/1999 H9N2	AJ404630, AJ404634, AJ404637, AJ404626, AJ404871, AJ404629, AJ404646, AJ404649
